# Berberine reduce allergic inflammation in a house dust mite allergic rhinitis mouse model

**DOI:** 10.1186/1939-4551-8-S1-A29

**Published:** 2015-04-08

**Authors:** Soo Whan Kim

**Affiliations:** 1 The Catholic University of Korea, South Korea

## Background

Berberine (Ber), is widely used as an antibacterial, antifungal, and anti-inflammatory drug, has been used as a gastrointestinal remedy for thousands of years in China. However, the direct evidence for the anti-inflammatory effects of Ber in allergic disease has remained elusive. The purpose of this study was to reveal whether Ber treatment reduces allergic inflammation in an AR mouse model and to elucidate the mechanisms.

## Methods

BALB/c mice were divided into control, Derf, Ber, Ber + Anti-CD25 groups. All mice except for the control group were sensitized by an i.p. injection of Dermatophagoides farinae (Derf). After sensitization, the Ber and the Ber+ Anti-CD25 groups were treated with Ber intranasally after challenge. The Ber+ Anti-CD25 groups were injected intraperitoneally with 250μg of anti-CD25 monoclonal antibody (mAb) a day before the first challenge. Allergic symptom scores, eosinophil counts and serum Derf-specific IgE levels were measured. T-bet, GATA-3, interferon-γ (IFN-γ), interleukin (IL)-10, IL-13 and Foxp3 mRNA expression were determined by real-time polymerase chain reaction and Western blot. CD4^+^CD25^+^Foxp3^+^ T cells were assessed by flow cytometry.

## Results

Symptom scores, serum Derf-specific IgE levels, GATA-3 mRNA levels, T-bet mRNA levels and tissue eosinophil counts were decreased in the Ber than the Derf groups (all, *p* < 0.05). In Ber + anti-CD25 mAb group, symptom scores, serum Derf-specific IgE levels, IL-13 mRNA levels, and IFN-γ mRNA levels were increased than control group. In Ber + anti-CD25 group, serum Derf-specific IgE levels, IL-13 mRNA levels and GATA-3 mRNA levels were increased than Ber groups. In Ber + anti-CD25 groups, serum IL-10 levels were decreased than control, Derf and Ber groups. In Ber +anti-CD25 mAb groups, Foxp3 mRNA levels were decreased than control group. In Ber group, Foxp3 mRNA levels were increased than control group. In Ber group, percentage of CD4+CD25+FOXP3 levels were increased than Derf group. In Ber + anti-CD25 mAb group, percentage of CD4+CD25+FOXP3 levels were decreased than Ber group. The percentage of CD4^+^CD25^+^Foxp3^+^ T cells were increased in the Ber than the Derf groups. And Foxp3 mRNA levels were increased in the Ber than control groups (all, *p* < 0.05).

## Conclusions

In our studies, Ber significantly reduces allergic inflammation. And our studies suggest that the mechanism of Ber may be associated with CD4+CD25+Foxp3+ Treg cells possibly through not only number but also the function.

